# Particularities of Urinary Tract Infections in Diabetic Patients: A Concise Review

**DOI:** 10.3390/medicina59101747

**Published:** 2023-09-29

**Authors:** Luminita-Georgeta Confederat, Mihaela-Iustina Condurache, Raluca-Elena Alexa, Oana-Maria Dragostin

**Affiliations:** 1 Department of Biomedical Sciences, “Grigore T. Popa” University of Medicine and Pharmacy of Iasi, 700115 Iasi, Romania; mihaela-iustina.condurache@umfiasi.ro; 2 “Sfântul Spiridon” County Emergency Clinical Hospital, 700111 Iasi, Romania; raluca-elena-alexa@email.umfiasi.ro; 3 Research Centre in the Medical-Pharmaceutical Field, Faculty of Medicine and Pharmacy, “Dunarea de Jos” University of Galati, 800008 Galati, Romania; oana.dragostin@ugal.ro

**Keywords:** diabetes, urinary tract infections, particularities, challenges, treatment

## Abstract

Diabetes mellitus is a chronic disease that, untreated or poorly controlled, can lead to serious complications, reducing life expectancy and quality. Diabetic patients are more likely to develop infections, including many common infections, but also pathognomonic ones such as emphysematous pyelonephritis, malignant otitis externa, mucormycosis and Fournier’s gangrene. Considering the fact that diabetic patients experience more frequently urinary tract infections (UTIs) with a worse prognosis than non-diabetic people, we conducted a review study based on data in the literature, following the particularities of UTIs in this group of patients, the risk factors, the mechanisms involved and the challenges in their management. The findings highlight that UTI in diabetic patients have some particularities, including a more frequent evolution to bacteremia, increased hospitalizations, and elevated rates of recurrence and mortality than non-diabetic patients. The possible risk factors identified seem to be female gender, pregnancy, older age, UTI in the previous six months, poor glycemic control and duration of diabetes. The mechanisms involved are related to glucosuria and bladder dysfunction, factors related to bacterial strains and host response. The bacterial strains involved in UTIs in diabetic patients and their antibiotic susceptibility profile are, with some exceptions, similar to those in non-diabetic people; however, the antimicrobial agents should be carefully chosen and the duration of the treatment should be as those required for a complicated UTI. The data related to the risk of developing UTIs in patients treated with SGLT-2 inhibitors, a new class of oral hypoglycaemic agents with cardiovascular and renal benefits, are controversial; overall, it was evidenced that UTIs occurred at the initiation of the treatment, recurrent infection was uncommon and the majority of UTIs responded to treatment with standard antibiotics. Moreover, interruption or discontinuation of SGLT-2 inhibitor as a result of UTI was rare and SGLT-2 inhibitors did not increase the risk of severe infections such as urosepsis and pyelonephritis.

## 1. Introduction

Diabetes has become a serious public health issue, being one of the most common chronic diseases causing life-threatening complications and reducing life expectancy and quality [1]. All data indicate that diabetes’ prevalence is increasing worldwide. The latest International Diabetes Federation (IDF) Diabetes Atlas reports that in 2021, the global prevalence of diabetes reached 10.5%, with about 44.7% of adults undiagnosed. This disease is considered “a pandemic of unprecedented magnitude”, affecting one of 10 adults worldwide [2]. According to recent IDF statistics, in 2021, there were about 536 million people with diabetes and the predictions for 2045 estimate that 783 million people will have diabetes, with the highest prevalence in the age group 75–79 years [3]. Concerning the distribution of diabetes subtypes in terms of prevalence, the statistical information for the year 2021 indicates that 96% of the total reported cases were attributed to type 2 diabetes. This phenomenon arises from the interplay of socioeconomic, demographic, environmental and genetic factors, including urbanization, aging of the population, decreased levels of physical activity and increased prevalence of being overweight and obesity [2,4]. A high proportion of diabetic patients (about 80%) are from low and middle-income countries. This trend is attributed to socioeconomic elements such as inadequate nutrition, poverty, and underdeveloped healthcare systems [4,5]. As expected, the number of people with diabetes living in urban areas is significantly higher than that of those living in rural areas (360 million vs. 176 million) and this number is expected to increase to 596 million in 2045, as a result of global urbanization with all its consequences [3].

Closely tied to prevalence statistics, and as an outcome of this dramatic increase of case numbers, diabetes represents a substantial burden to healthcare systems. According to the Global Burden of Disease Study 2021, the expenditures related to diabetes were US$966 billion globally, and these are expected to reach to $1054 billion by 2045 [4,6].

**Figure 1:**
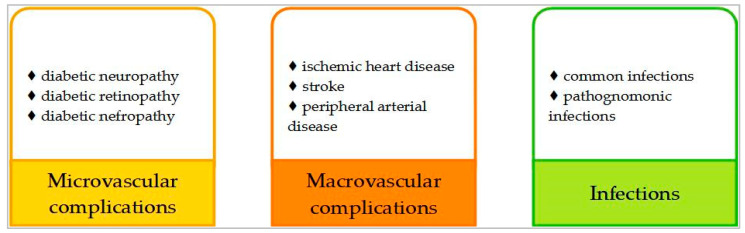
Complications of diabetes.

Undiagnosed and poorly controlled diabetes can lead or significantly contribute to serious life-threatening complications, such as heart attack, stroke, kidney failure, blindness and lower-limb amputations, resulting in reduced quality of life and increased healthcare costs [2]. Additionally, many observational and some interventional studies, such as “Action in Diabetes and Vascular Disease” (ADVANCE), “Veterans Affairs Diabetes Trial” (VADT), “Action to Control Cardiovascular Risk in Diabetes” (ACCORD), and “Empagliflozin Cardiovascular Outcome Event” (EMPA-REG OUTCOME) demonstrated that in type 2 diabetes, long-term glucose variability, defined as fluctuations outside of the recommended range in successive measures, is correlated with an increased risk of both microvascular and macrovascular complications [7,8,9,10,11]. Figure 1 illustrates the systematization of diabetes’ complications. 

The global epidemic of prediabetes and diabetes has led to a corresponding epidemic of related complications [12]. Among these complications, diabetic neuropathy, defined as a group of syndromes caused by damage to the peripheral and autonomic nervous systems, is the most prevalent, occurring in up to 50% of people with diabetes [13]. The most common form of diabetic neuropathy, distal symmetric polyneuropathy, is associated with a loss of sensory function, pain, numbness and reduced quality of life [14]. Autonomic neuropathies, including cardiac autonomic neuropathy, gastrointestinal dysmotility, diabetic cystopathy and impotence, involve serious medical consequences and reduce the quality of life. Diabetic retinopathy is a common complication of diabetes and remains one of the leading causes of preventable blindness. In the Global Burden of Disease Study, diabetic retinopathy was the fifth cause of blindness and severe vision impairment [15,16]. A systematic review of 59 population-based studies found that the global prevalence of diabetic retinopathy was 22.2% [15]. Diabetic nephropathy, characterized by persistent albuminuria and progressive decline in renal function, is a chronic complication found in 20% to 50% of diabetic patients and is the most common cause of end-stage kidney disease that requires dialysis or renal transplantation. Additionally, the outcome of both type 1 and type 2 diabetic patients who develop nephropathy is significantly worse than for people without this complication [17].

Concerning macrovascular complications, it is well established that cardiovascular complications of diabetes represent the leading cause of mortality and disability, being responsible for 80% of diabetic mortality [18]. Traditionally, the prevention and management of macrovascular complications of diabetes have been focused on atherosclerotic cardiovascular disease, including ischemic heart disease, stroke and peripheral vascular disease. However, a consensus report of the American Diabetes Association consider heart failure an underappreciated complication of diabetes, which may develop in people with diabetes even in the absence of hypertension, dyslipidemia, or coronary or valvular heart disease [19,20]. Additionally, nonalcoholic fatty liver disease (NAFLD), which is strongly associated with type 2 diabetes mellitus, is considered an independent risk factor for cardiovascular disease [21].

Another complication of diabetes that significantly contributes to the global burden of disability is diabetic foot; the lifetime risk of developing this complication seems to be between 19% and 34%, and recurrence is common after initial healing [22]. Neuropathy and ischaemia—in the form of peripheral arterial disease—are the two major pathogenic mechanisms of the diabetic foot, which lead to foot ulceration and Charcot neuroartropathy; these can be complicated by infections and eventually may result in amputations [22].

When addressing the complications of diabetes, chronic microvascular and macrovascular complications presented above are widely discussed; the risk of infections in diabetic patients in the broad context of diabetes complications is underestimated, even if the association between these medical conditions is accepted clinically. Diabetic patients are more likely to develop common infectious diseases, urinary tract infections being the most prevalent type of infection. The main topic of this review is data in the literature on the particularities of UTIs in diabetic patients, a current topic of particular importance, involving risk factors, prognosis and challenges in their management. 

## 2. Infections in Patients with Diabetes

Diabetes seems to increase the risk of infections through different pathways as impaired immune responses within the hyperglycemic environment. Additionally, common complications of diabetes include neuropathy and vascular insufficiency [23,24]. Related to this, a retrospective cohort study including 102,493 patients showed that compared with control subjects without diabetes, patients with diabetes had higher rates of all types of infections. During the follow-up period, 56.9% of patients with type 2 diabetes and 55% of patients with type 1 diabetes had at least one infection, compared with 46.2% of control subjects [23]. Additionally, patients with diabetes have a worse evolution of infectious diseases reflected by increased rates of hospital admission, a longer period of hospitalization and more complications [25]. The same retrospective cohort study estimated that 6% of infection-related hospitalizations and 12% of infection-related deaths were attributable to diabetes [23]. Another study from the USA estimated that 10% of Emergency Department presentations of diabetic patients were for infections and people with diabetes had a two times higher risk of being hospitalized for an infection than people without diabetes [26]. The risk factors for development of infections in diabetic patients and for poor outcomes were represented by age (over 70 years), higher fasting plasma glucose and HbA1c levels, obesity, hypertriglyceridemia, smoking, increased levels of serum creatinine, longer duration of diabetes, and the presence of diabetes specific complications [23,27]. Concerning the type of infections, individuals with diabetes present an elevated susceptibility to various prevalent infections like urinary tract infections (UTIs), respiratory tract infections, and skin and soft tissue infections but there are also rare infections, often pathognomonic for a patient having diabetes, such as emphysematous pyelonephritis, malignant otitis externa, mucormycosis and Fournier’s gangrene [24,28].

## 3. Urinary Tract Infections in Diabetic Patients

### 3.1. Prevalence

Several studies evaluated UTI as a potential complication of type 2 diabetes, being widely accepted that this type of infection is more commonly experienced by people with diabetes than those without diabetes [29]. Additionally, diabetic patients have bacteraemia more often, with the urinary tract as the most common site for these infections, as well as higher mortality rates [30].

A large, retrospective cohort study including 179,580 subjects with type 2 diabetes, showed that UTIs were more prevalent in diabetic patients (9.4% vs. 5.7%); related to the type of UTI, both cystitis and pyelonephritis were more common in diabetic patients than in people without diabetes (1.34% vs. 0.9% for cystitis and 0.14% vs. 0.07% for pyelonephritis, respectively). Concerning gender distribution, as expected, a high proportion of women experienced all forms of UTI (14% vs. 5%). Additionally, recurrence of UTI was higher in subjects with diabetes (1.6% vs. 0.6%) [29].

A systematic review showed that the prevalence of asymptomatic bacteriuria (ASB), defined as two consecutives urine specimens from which the same bacterial strain was isolated in quantitative counts ≥ 10^5^ UFC/mL, from a patient without UTI symptoms, was 12.2% in the case of patients with diabetes vs. 4.5% in patients without diabetes. The prevalence of ASB was higher in both women (14.2% vs. 5.1%) and men (2.3% vs. 0.8%) with diabetes compared with healthy control, respectively [31].

In a Spanish prospective study including patients with bacteraemia, a comparison was made between individuals with diabetes and those without diabetes; the results showed that diabetic patients had bacteraemia more often and the urinary tract was the most common location; the incidence rates per 1000 admissions comparing diabetic vs. non-diabetic patients were 26.8 vs. 15.5, respectively, for bacteraemia and 8.7 vs. 2.2, respectively, for urinary tract source of bacteraemia [30].

A UK-based observational study evidenced a nearly 60% increase in the risk of developing urinary tract infections among patients with type 2 diabetes, with the possible risk factors being female gender, pregnancy, older age, UTI in the previous six months, and poor glycaemic control [32]. Another study including 1157 Indian patients, showed a correlation between the percentage of patients with UTI and the duration of diabetes (41.8% < 10 years vs. 58.2% > 10 years) and glycemic control (19.3%—HbA1c < 8%, 33.2%—HbA1c: 8–9%, 64.9%—HbA1c > 9%) [33]. By contrast, Miftode et al. conducted a retrospective cohort study that enrolled 354 patients with confirmed UTIs whose results showed that some traditional risk factors, such as diabetes or cardiovascular diseases, were not significantly correlated with a poor UTI outcome [34].

### 3.2. Mechanisms Involved

**Figure 2:**
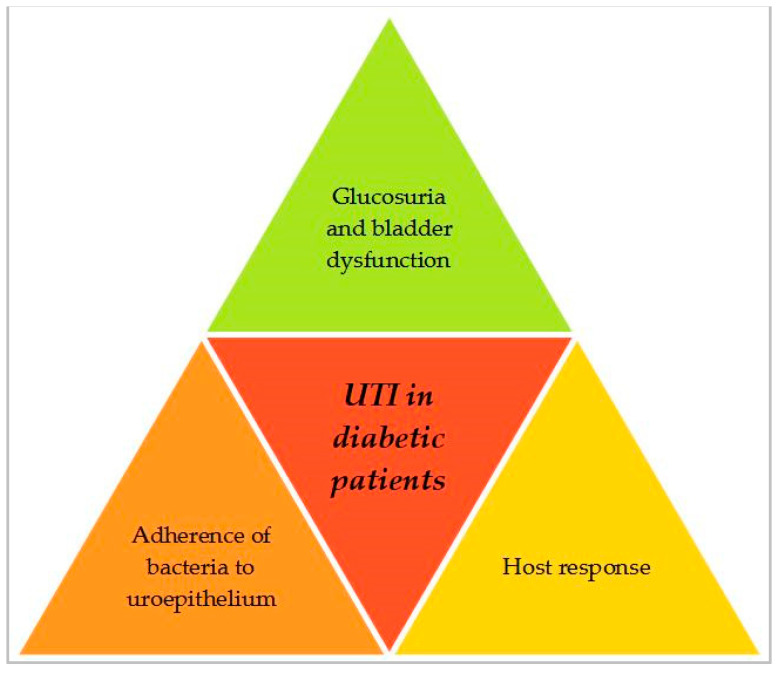
Main mechanisms involved in the development of UTI in diabetic patients.

The increased prevalence of UTIs in diabetic people may result from variations in the host response between diabetic and non-diabetic patients, disparities in the infecting microbial strains, or a combination of both factors. While the exact mechanisms remain partially elucidated, a number of potential hypotheses have been suggested to clarify the connection between diabetes and UTI, including altered growth conditions (resulting from glucosuria and diabetes associated neuropathy) and altered pathogen–host interactions as a result of diabetes [35]. Figure 2 illustrates the mechanisms involved in the development of UTIs in diabetic patients, which will be concisely discussed below.

#### 3.2.1. Glucosuria and Bladder Dysfunction

As was presented in the Introduction section, diabetic cysthopathy is a form of diabetic autonomic neuropathy resulting in damage to the genitourinary system and leading to dysfunctional bladder, thereby creating the opportunity for the development of urinary tract infections. Additionally, higher urinary glucose concentrations as a consequence of poor glycemic control create a favorable environment for bacterial multiplication [30]. Related to this, a study reported that urine samples containing glucose in concentrations equivalent to moderate and severe glucosuria significantly enhanced bacterial growth after six hours, compared with normal urine [35]. Furthermore, in a clinical study, the association between glucosuria and ASB was evidenced, the urinary glucose concentration measuring approximately 352.2 mg/dL in diabetes patients with ASB compared to about 62.4 mg/dL in patients without ASB [36]. By contrast, in a large cohort study including 636 women with diabetes, there was no association between glucosuria and ASB; 42% of the women without ASB and 38% with ASB presented glucosuria, suggesting that probably there is a threshold urinary glucose concentration that determines ASB [37]. Another study including 348 women with type 2 diabetes reported the presence of ASB at baseline as a risk factor for the development of UTI, but glucosuria was not associated with the development of symptomatic UTI [35].

#### 3.2.2. Adherence of Bacteria to the Uroepithelium

Another aspect that could bring a contribution to the pathogenesis of UTI and ASB is the fact that in patients with diabetes, an increased adherence of bacterial strains to the uroepithelial cells was observed [38]. This phenomenon could be applicable to all Gram-negative uropathogens responsible for UTI in diabetic patients (*Klebsiella* spp., *Enterobacter* spp., *Proteus* spp., *Pseudomonas* spp.), but the mechanisms of adherence were studied especially in *E. coli* expressing type-1 fimbriae—the virulence factor that plays an important role in the pathogenesis of UTI. This increased adherence was positively correlated with HbA1c values, indicating that poorly controlled diabetic people had a higher adherence of *E. coli* to uroepithelial cells compared with well-controlled diabetic patients [35]. The difference in adherence to uroepithelial cells has been hypothesized to stem from variations in receptors for type-1 fimbriae between individuals with and without diabetes, resulting from altered glycosylation of uroplakins, the major glycoproteins of the bladder mucosa [35].

#### 3.2.3. Host Response

It was well established that the hyperglycaemic environment is responsible for altering immune function in patients with diabetes, affecting several aspects of immunity, such as polymorphonuclear leukocyte function and adhesion, chemotaxis and phagocytosis, thus, contributing to the pathogenesis of UTI. Additionally, it was demonstrated that in women with diabetes and ASB, there were lower urinary interleukin (IL-6 and IL-8) concentrations compared with non-diabetic women with ASB, being correlated with a lower leukocyte count that plays a role in the pathogenesis of ASB and UTI in diabetic patients [35,39]. Considering the well-established relationship between the hyperglycaemic environment and the alteration of the immune response, the improvement of glycaemic control in diabetic patients seems to be of particular importance in the prevention of UTIs; additionally, maintaining optimal glycaemic values during the treatment of an UTI could be associated with a better evolution of the infection. 

### 3.3. Microorganism Involved and Antibiotic Susceptibility Profile

Some studies showed that the microorganisms involved in UTIs in diabetic patients are comparable with those found in non-diabetic patients with complicated urinary tract infections, with the mention that bacterial strains other than *E. coli* are more frequently isolated from the urine of diabetic patients than from non-diabetic patients [30]. The most common pathogens are *E. coli*, *Klebsiella* spp., *S. aureus*, *Enterobacter* spp., *Proteus* spp., *Pseudomonas* spp., Group B *Streptococci* and *Enterococcus faecalis* [40,41,42].

There are not published randomized trials that could answer the question concerning the optimal duration of the treatment of UTI in diabetic patients; however, these infections should be considered as complications for UTIs and should be treated for a period of 7–14 days [30].

The data concerning the increase in antimicrobial resistant strains in diabetic patients with UTI are controversial. 

Some studies suggest that diabetes mellitus per se does not influence the susceptibility patterns of uropathogens to antimicrobials and, with some few exceptions, there have been no differences between the antimicrobial susceptibility profile of bacterial strains isolated from diabetic and non-diabetic patients [43]. Meiland et al. have reported *E. coli* strains isolated from the urine of diabetic women to have lower antimicrobial resistance rates than those isolated from the samples of non-diabetic women. Also, Bonadio et al. found that *E. coli* strains isolated from diabetic and non-diabetic patients have similar rates of resistance to ampicillin, cotrimoxazole, ciprofloxacin and nitrofurantoin [44,45]. The study conducted by Wang et al. on 271 *E. coli* strains isolated from urine samples (190 from diabetic and 81 from non-diabetic patients) reported a similar antimicrobial resistance profile between diabetic and non-diabetic patients, excepting a higher resistance rate to the second- and third-generation cephalosporins in the diabetic patients [44]. In addition, Owusu et al. conducted a prospective cross-sectional study including 100 diagnosed diabetic patients and 100 non-diabetic individuals that showed that bacterial strains isolated from both diabetic and non-diabetic individuals were highly susceptible to most of the antibiotics tested, especially nitrofurantoin, cefuroxime, ceftriaxone, and cefotaxime [46].

On the other hand, there are studies that evidence a different susceptibility profile of bacterial strains isolated from diabetic patients. Asghar et al. conducted a retrospective study on 222 patients that showed that *E. coli* isolated from diabetic patients was mainly susceptible to aminoglycosides and fosfomycin, having higher resistance rates to ampicillin and third-generation cephalosporins as well as *K. pneumoniae* showing higher resistance rates to cephalosporins and ciprofloxacin [47]. Also, Paudel el al. conducted a systematic review that showed that diabetic individuals are at >2-fold higher risk of UTI by drug-resistant uropathogens: *E. coli* isolates from diabetic patients with UTIs were multidrug resistant and diabetes increased the risk of UTIs with extended spectrum cephalosporin-resistant *Enterobacteriaceae* and extended spectrum β-lactamase (ESBL) producing *E. coli* and *K. pneumoniae*; diabetes increases the risk of UTI by uropathogens resistant to quinolone antibiotics [48,49,50]. In addition, Malmartel et al. conducted a cross-sectional study that compared bacterial resistance rates to ofloxacin, cefixim, cotrimoxazole, nitrofurantoin and fosfomycin in UTIs between patients with and without diabetes; after controlling for variables such as sex, age, and prior UTI history, the findings indicated elevated rates of resistance to ofloxacin and cefixime in patients with diabetes [42].

Taking into account these data, the choice of treatment for UTI could be the same in diabetic and non-diabetic individuals, depending on the local resistance patterns of the most commonly found pathogens. In addition, considering that cotrimoxazole is an antimicrobial agent associated with the potential to induce hypoglycemia, it is advised not to prioritize it as the initial treatment option for individuals with diabetes [30].

Closely related to treatment, it is of particular importance to prevent UTI with resistant strains and the development of antibiotic resistance. Firstly, nonpharmacological measures, such as hygiene and good hydration in order to help the elimination of bacteria, should be respected. Then, it is important to differentiate ASB from UTIs and to consult an infectionist to determine whether antibiotherapy is recommended. Finally, the choice of the antimicrobial agents should be based on the result of laboratory susceptibility test, the features of the patient and the local resistance patterns, in the cases where empirical antibiotherapy is required. 

### 3.4. Challenges in the Management of Urinary Tract Infections in Diabetic Patients—SGLT-2 Inhibitors and the Risk of Urinary Tract Infections

Sodium–glucose co-transporter 2 (SGLT-2) inhibitors represent a relatively new class of oral antidiabetic agents that reduce serum glucose levels by inhibiting glucose reabsorption in the proximal tubule, thus, inducing renal glucosuria [51]. In addition to the antihyperglycemic effect, this class of drugs demonstrated reductions in cardiovascular events and mortality as well as renal protection [52,53]. Taking into account that SGLT-2 inhibitors increase urinary glucose concentration, thus providing substrate for bacteria to grow, this class of drug could potentially increase the risk for uro-genital infections [54,55]. Nonetheless, outcomes of the studies conducted in this context are marked by conflicting findings.

Pooled data from 12 double-blind controlled clinical trials with dapagliflozin evaluated the effect of pharmacologically induced glucosuria on the incidence of UTI and evidenced that that rates of clinically diagnosed UTI were slightly higher in patients treated with dapagliflozin compared with placebo, this type of infections being more frequent in women. It was noticed that the first UTI events occurred at the initiation of the treatment and recurrent infection was uncommon. However, severe urinary tract infections such as pyelonephritis were infrequent in both groups (dapagliflozin and placebo) and the majority of UTIs responded to treatment with standard antibiotics. Moreover, interruption or discontinuation of SGLT-2 inhibitor as a result of UTI was rare (0.2–0.3% of patients treated with dapagliflozin compared with 0.1% in the placebo group [35]. Purkin et al. found that dapagliflozin 10 mg daily was associated with a significant risk of UTI compared with placebo, but SGLT-2 inhibitors did not increase the risk of severe infections, such as urosepsis and pyelonephritis [56].

Concerning other SGLT-2 inhibitors, there are less data in the literature; it is important to note that infections were not recorded using a consistent methodology and it is difficult to compare infection rates between different agents. A phase 2 study involving canagliflozin aimed to evaluate the prevalence of ASB and the incidence of UTI in patients with type 2 diabetes; the initial prevalence of ASB was similar between the canagliflozin group and placebo (6.4% vs. 6.5%), and after 12 weeks of treatment there were no obvious differences between the two groups. The incidence of UTI occurred in 5% in the canagliflozin group vs. 3.8% in the placebo group [35]. Given that some studies suggest that long-term use of canaglifozin is associated with uro-genital infcetions, a large, randomised, double-blind study compared canagliflozin with glimepiride in patients poorly controlled with metformin; the results showed modest elevations in UTI rates in canagliflozin vs. glimepiride (6% vs. 5%). Related to empagliflozin, a 12-week study including 495 type 2 diabetic patients inadequately controlled with metformin found similar rates of UTI in the empagliflozin and sitagliptin groups (4.0% vs. 4.2%) but higher rates than in the placebo group (2.8%) [35]. 

By contrast, population-based cohort studies evidenced no statistical association between the use of SGLT-2 inhibitors and the risk of UTI. A larger retrospective population-based study conducted in Canada showed that SGLT-2 inhibitors were not statistically associated with increased risk of UTI and that the history of UTI in the last 60 days did not increase the rate of UTI in patients using SGLT-2 inhibitors [57]. Another study conducted by Dave et al., which assessed if patients initiating SGLT-2 inhibitors were at an increased risk of developing severe UTI (hospitalization for UTI, urinary sepsis and pyelonephritis) compared with dipeptidyl peptidase-4 (DPP-4) inhibitors and glucagon-like peptide 1 receptor agonists (GLP-1 RA), showed that the risk of severe and non-severe UTI among patients initiating SGLT-2 inhibitors was similar to the rates among patients initiating other classes of drugs [58]. Finally, Alkabbani et al. showed, in a large population-based cohort study, that SGLT-2 inhibitors were not associated with a higher risk of UTI than DPP-4 inhibitors, GLP-1 RA, or tiazolidindiones; however, the risk of UTI was lower when compared with insulin treatment [59].

## 4. Conclusions

When addressing the complications of diabetes, the risk of infections is often underestimated, even if it is well known that people with diabetes are at higher risk of developing different types of infections compared with non-diabetic people. Urinary tract infections are commonly experienced by diabetic people, with more frequent evolution to bacteraemia, more hospitalizations and, often, a higher recurrence and mortality rate than non-diabetic patients. Even if the bacterial strains isolated from the urine of diabetic patients with UTI are similar to those isolated from non-diabetic people, these infections should be considered as complicated UTIs and treated for 7–14 days. The conclusions of the studies that followed the differences between antibiotic resistance rates of the strains isolated from diabetic patients compared with non-diabetic are controversial; thus, the choice of the antimicrobial agents should be based on the results of laboratory susceptibility test, the features of the patient and the local resistance patterns, if the antibiotherapy is urgently needed. Finally, there were many debates around the risk of urogenital infections associated with SGLT-2 inhibitors; theoretically this side effect is plausible and explainable through their mechanism of action, but large cohort studies showed that the risk is not higher compared to other new drug classes. 

## Data Availability

Not applicable.

## References

[1] Healda A.H., Stedmanc M., Daviesc M., Livingstond M., Alshamesb R., Lunte M., Raymanf G., Gadsbyg R. (2020). Estimating life years lost to diabetes: Outcomes from analysis of National Diabetes Audit and Office of National Statistics data. Cardiovasc. Endocrinol. Metab..

[2] International Diabetis Federation (2021). Diabetes is ‘‘a pandemic of unprecedented magnitude’’ now affecting one in 10 adults worldwide. Diabetes Res. Clin. Pract..

[3] Sun H., Saeedi P., Karuranga S., Pinkepank M., Ogurtsova K., Duncan B.B., Stein C., Basit A., Chan J.K.N., Mbanya J.C. (2022). IDF Diabetes Atlas: Global, regional and country-level diabetes prevalence estimates for 2021 and projections for 2045. Diabetes Res. Clin. Pract..

[4] GBD 2021 Diabetes Collaborators (2023). Global, regional, and national burden of diabetes from 1990 to 2021, with projections of prevalence to 2050: A systematic analysis for the Global Burden of Disease Study 2021. Articles.

[5] Arhire L.I. (2015). Orthorexia Nervosa: The unhealthy obssesion for healthy food. Rev. Med. Chir..

[6] Dieleman J.L., Cao J., Chapin A., Chen C., Li Z., Liu A., Horst C., Kaldjian A., Matyasz T., Scott K.W. (2020). US Health Care Spending by Payer and Health Condition, 1996–2016. JAMA.

[7] Ceriello A., Prattichizzo F. (2021). Variability of risk factors and diabetes complications. Ceriello Prattichizzo Cardiovasc. Diabetol..

[8] Hirakawa Y., Arima H., Zoungas S., Ninomiya T., Cooper M., Hamet P., Mancia G., Poulter N., Harrap S., Woodward M. (2014). Impact of Visit-to-Visit Glycemic Variability on the Risks of Macrovascular and Microvascular Events and All-Cause Mortality in Type 2 Diabetes: The ADVANCE Trial. Diabetes Care.

[9] Zhou J.J., Schwenke D.C., Bahn G., Reaven P. (2018). Glycemic Variation and Cardiovascular Risk in the Veterans Affairs Diabetes Trial. Diabetes Care.

[10] Sheng C.S., Tian J., Miao Y., Cheng Y., Yang Y., Reaven P.D., Bloomgarden Z.T., Ning G. (2020). Prognostic Significance of Long term HbA1c Variability for All Cause Mortality in the accord trial. Diabetes Care.

[11] Ceriello A., Ofstad A.P., Zwiener I., Kaspers S., George J., Nicolucci A. (2020). Empaglifozin reduced long-term HbA1c variability and cardiovascular death: Insights from the EMPA-REG OUTCOME trial. Cardiovasc. Diabetol..

[12] Feldman E.L., Callaghan B.C., Pop-Busui R., Zochodne D.H., Wright D.E., Bennett D.L., Bril V., Russell J.W., Viswanathan V. (2019). Diabetic neuropathy. Nat. Rev..

[13] Callaghan B.C., Price R.S., Chen K.S., Feldman E.L. (2015). Peripheral neuropathy: The importance of rare subtypes. JAMA Neurol..

[14] Pop-Busui R., Boulton A.J.M., Feldman E.L., Bril V., Freeman R., Malik R.A., Sosenko J.M., Ziegler D. (2017). Diabetic Neuropathy: A Position Statement by the American Diabetes Association. Diabetes Care.

[15] Teo Z.L., Tham Y.C., Yu M., Chee M.L., Rim T.N., Cheung N., Bikbov M.M., Wang Y.X., Tang Y., Lu Y. (2021). Global Prevalence of Diabetic Retinopathy and Projection of Burden through 2045. Systematic Review and Meta-analysis. Ophthalmology.

[16] GBD 2019 Blindness and Vision Impairment Collaborators^*^ and Vision Loss Expert Group of the Global Burden of Disease Study (2021). Causes of blindness and vision impairment in 2020 and trends over 30 years, and prevalence of avoidable blindness in relation to VISION 2020: The Right to Sight: An analysis for the Global Burden of Disease Study. Lancet Glob. Health.

[17] Selby N.M., Taal M.W. (2020). An updated overview of diabetic nephropathy: Diagnosis, prognosis, treatment goals and latest guidelines. Diabetes Obes. Metab..

[18] Ahmad M.N., Farah A.I., Al-Qirim T.M. (2020). The cardiovascular complications of diabetes: A striking link through protein glycation. Rom. J. Int. Med..

[19] Doumas M., Imprialos K., Stavropoulos K., Athyros V.G. (2020). Pharmacological Management of Type 2 Diabetes Complications. Curr. Vasc. Pharmacol..

[20] Pop-Busui R., Januzzi J.L., Bruemmer D., Butalia S., Green J.B., Horton W.B., Knight C., Levi M., Rasouli N., Richardson C.R. (2022). Heart Failure: An Underappreciated Complication of Diabetes. A Consensus Report of the American Diabetes Association. Diabetes Care.

[21] Gavril R.S., Mitu F., Leon M.M., Mihalache L., Arhire L.I., Grosu C., Gherasim A., Nita O., Uugureanu I.O., Oprescu A.C. (2016). Biomarkers of Inflammation in Patients with Type 2 Diabets Mellitus and Hepatic Steatosis. Rev. Chim..

[22] Edmonds M., Manu C., Vas P. (2021). The current burden of diabetic foot disease. J. Clin. Orthop. Trauma..

[23] Carey I.M., Critchley J.A., DeWilde S., Harris T., Hosking F.J., Cook D.J. (2018). Risk of Infection in Type 1 and Type 2 Diabetes Compared With the General Population: A Matched Cohort Study. Diabetes Care.

[24] Pearson-Stuttard J., Blundell S., Harris T., Cook D.G., Critchley J. (2016). Diabetes and infection: Assessing the association with glycaemic control in population-based studies. Lancet Diabetes Endocrinol..

[25] Suaya J.A., Eisenberg D.F., Fang C., Miller L.G. (2013). Skin and Soft Tissue Infections and Associated Complications among Commercially Insured Patients Aged 0–64 Years with and without Diabetes in the U.S. PLoS ONE.

[26] Korbel L., Spencer J.D. (2015). Diabetes Mellitus and Infection: An Evaluation of Hospital Utilization and Management Costs in the United States. J. Diabetes Complicat..

[27] Masoodi S.R., Wani A.I., Misgar R.A., Gupta V.K., Bashir M.I., Zargar A.H. (2007). Pattern of infections in patients with diabetes mellitus-Data from a tertiary care medical centre in Indian sub-continent. Diabetes Metab. Syndr. Clin. Res. Rev..

[28] Cooke F. (2019). Infections in people with diabetes. Medicine.

[29] Fu A.Z., Iglay K., Qiu Y., Engel A., Shanka R., Brodovicz K. (2014). Risk characterization for urinary tract infections in subjects with newly diagnosed type 2 diabetes. J. Diabetes Its Complicat..

[30] Geerlings S.E. (2008). Urinary tract infections in patients with diabetes mellitus: Epidemiology, pathogenesis and treatment. Int. J. Antimicrob. Agents.

[31] Renko M., Tapanainen P., Tossavainen P., Pokka T., Uhari M. (2011). Meta-Analysis of the Significance of Asymptomatic Bacteriuria in Diabetes. Diabetes Care.

[32] Hirji I., Guo Z., Andersson S.W., Hammar N., Gomez-Caminero A. (2012). Incidence of urinary tract infection among patients with type 2 diabetes in the UK General Practice Research Database (GPRD). J. Diabetes Its Complicat..

[33] Janifer J., Geethalakshmi S., Satyavani K., Viswanathan V. (2009). Prevalence of lower urinary tract infection in South Indian type 2 diabetic subjects. Indian. J. Nephrol..

[34] Miftode I.L., Pasare M.A., Miftode R.S., Nastase E., Plesca C.E., Lunca C., Miftode E.G., Timpau A.S., Iancu L.S., Dorneanu O.S. (2022). What doesn’t kill them makes them stronger: The impact of the resistance patterns of urinary *Enterobacterales* isoaltes in patients from a tertiary hospital in Eastern Europe. Antibiotics.

[35] Geerlings S., Fonseca V., Castro-Diaz D., List D., Parikh S. (2014). Genital and urinary tract infections in diabetes: Impact of pharmacologically-induced glucosuria. Diabetes Res. Clin. Pract..

[36] Turan H., Serefhanoglu K., Torun A.N., Kulaksizoglu S., Kulaksizoglu M., Pamuk P., Arslan H. (2008). Frequency, risk factors, and responsible pathogenic microorganisms of asymptomatic bacteriuria in patients with type 2 diabetes mellitus. Jpn. J. Infect. Dis..

[37] Geerlings S.E., Stolk R.P., Camps M.J.L., Netten P.M., Hoekstra J.B.L., Bouter P., Bravenboeur B., Collet J.T., Jansz A.R., Hoepelman A.I.M. (2000). Asymptomatic Bacteriuria May Be Considered a Complication in Women With Diabetes. Diabetes Care.

[38] Geerlings S.E., Meiland R., Van Lith E.C., Brouwer E.C., Gaastra W., Hoepelman A.I.M. (2002). Adherence of Type 1–Fimbriated Escherichia coli to Uroepithelial Cells. Diabetes Care.

[39] Hoepelman A.I.M., Meiland R., Geerlings S.M. (2003). Pathogenesis and management of bacterial urinary tract infections in adult patients with diabetes mellitus. Int. J. Antimicrob. Agents.

[40] Hamdan H.Z., Kubbara E., Adam A.M., Hassan O.S., Suliman S.O., Adam I. (2015). Urinary tract infections and antimicrobial sensitivity among diabetic patients at Khartoum, Sudan. Ann. Clin. Microbiol. Antimicrob..

[41] Niranjan V., Malini A. (2014). Antimicrobial resistance pattern in Escherichia coli causing urinary tract infection among inpatients. Indian. J. Med. Res..

[42] Malmartel A., Ghasarossian C. (2016). Bacterial resistance in urinary tract infections in patients with diabetes matched with patients without diabetes. J. Diabetes Its Complicat..

[43] Ghenghesh K.S., Elkateb E., Berbash N., Abdel Nada R., Ahmed S.F., Rahouma A., Seif-Enasser N., Elkhabroun M.A., Belresh T., Klena J.D. (2009). Uropathogens from diabetic patients in Libya: Virulence factors and phylogenetic groups of Escherichia coli isolates. J. Med. Microbiol..

[44] Wang M.K., Tseng C.C., Wu A.B., Lin W.H., Teng C.H., Yan J.J., Wu J.J. (2013). Bacterial characteristics and glycemic control in diabetic patients with Escherichia coli urinary tract infection. J. Microbiol. Immunol. Infect..

[45] Bonadio M., Costarelli S., Morelli G., Tartaglia T. (2006). The influence of diabetes mellitus on the spectrum of uropathogens and the antimicrobial resistance in elderly adult patients with urinary tract infection. BMC Infect. Dis..

[46] Owusu E., Adjei H., Afutu E. (2022). Similarities in Bacterial Uropathogens and Their Antimicrobial Susceptibility Profile in Diabetics and Their Non-Diabetic Caregivers at a National Diabetes Management and Research Centre, Accra-Ghana. Diseases.

[47] Asghar M.S., Akram M., Singh M., Yasmin F., Yaseen R., Ahmed N., Siddiqui M., Hassan M., Rasheed U., Ali A. (2021). Characteristics of Asymptomatic Bacteriuria in Diabetes Mellitus Patients: A Retrospective Observational Study. Cureus.

[48] Paudel S., John P.P., Poorbaghi S.L., Randis T.M., Kulkarni R. (2022). Systematic Review of Literature Examining Bacterial Urinary Tract Infections in Diabetes. J. Diabetes Res..

[49] Anesi J.A., Lautenbach E., Garrigan I.N.C., Bilker W.B., Wheeler M., Tolomeo P., Han J.H. (2016). Clinical and Molecular Characterization of Community-Onset Urinary Tract Infections Due to Extended-Spectrum Cephalosporin-Resistant Enterobacteriaceae. Infect. Control Hosp. Epidemiol..

[50] Briongos-Figuero L.S., Gómez-Traveso T., Bachiller-Luque P., Domínguez-Gil González M., Gómez-Nieto A., Palacios-Martín T., González-Sagrado M., Dueñas-Laita A., Pérez-Castrillón J.L. (2012). Epidemiology, risk factors and comorbidity for urinary tract infections caused by extended-spectrum beta-lactamase (ESBL)-producing enterobacteria. Int. J. Clin. Pr..

[51] Vallon V. (2015). The Mechanisms and Therapeutic Potential of SGLT2 Inhibitors in Diabetes Mellitus. Annu. Rev. Med..

[52] Neal N., Perkovic V., Mahaffey K.W., de Zeeuw D., Fulcher G., Erondu N., Shaw W., Law G., Desai M., Matthews D.R. (2017). Canagliflozin and Cardiovascular and Renal Events in Type 2 Diabetes. N. Engl. J. Med..

[53] Patorno E., Goldfine A.B., Schneeweiss S., Everett B.M., Glynn R.J., Liu J., Kim S.C. (2018). Cardiovascular outcomes associated with canagliflozin versus other non-gliflozin antidiabetic drugs: Population based cohort study. BMJ..

[54] Vasilakou D., Karagiannis T., Athanasiadou E., Mainou M., Liakos A., Bekiari E., Sarigianni M., Matthews D.R., Tsapas A. (2013). Sodium-glucose cotransporter 2 inhibitors for type 2 diabetes: A systematic review and meta-analysis. Ann. Intern. Med..

[55] Li D., Wang T., Shen S., Fang Z., Dong Y., Tang H. (2017). Urinary tract and genital infections in patients with type 2 diabetes treated with sodium-glucose co-transporter 2 inhibitors: A meta-analysis of randomized controlled trials. Diabetes Obes. Metab..

[56] Puckrin R., Saltiel M.P., Reynier P., Azoulay L., Yu O.H.Y., Filion K.B. (2018). SGLT-2 inhibitors and the risk of infections: A systematic review and meta-analysis of randomized controlled trials. Acta Diabetol..

[57] Kamei J., Yamamoto S. (2021). Complicated urinary tract infections with diabetes mellitus. J. Infect. Chemother..

[58] Dave C.V., Schneeweiss S., Kim D., Fralick M., Tong A., Patorno E. (2019). Sodium-glucose cotransporter 2 inhibitors and the risk of severe urinary tract infections. Ann. Intern. Med..

[59] Alkabbani W., Zongo A., Minhas-Sandhu J.K., Eurich D.T., Shah B.R., Alsabbagh W., Gamble J.M. (2022). Sodium-Glucose Cotransporter-2 Inhibitors and Urinary Tract Infections: A Propensity Scoreematched Population-based Cohort Study. Can. J. Diabetes.

